# The Economic Burden of Pneumococcal Disease in Children: A Population-Based Investigation in the Veneto Region of Italy

**DOI:** 10.3390/children9091347

**Published:** 2022-09-03

**Authors:** Elisa Barbieri, Gloria Porcu, Tanaz Petigara, Francesca Senese, Gian Marco Prandi, Antonio Scamarcia, Luigi Cantarutti, Anna Cantarutti, Carlo Giaquinto

**Affiliations:** 1 Division of Pediatric Infectious Diseases, Department of Women’s and Children’s Health, University of Padova, 35131 Padova, Italy; 2 Unit of Biostatistics Epidemiology and Public Health, Department of Statistics and Quantitative Methods, University of Milano-Bicocca, 20126 Milan, Italy; 3 Center for Observational and Real-World Evidence, Merck & Co., Inc., Rahway, NJ 07065, USA; 4 MSD Italy, 00189 Rome, Italy; 5 Pedianet Project, 35138 Padova, Italy

**Keywords:** pneumococcal disease, pneumonia, acute otitis media, invasive pneumococcal disease, economic burden, healthcare resource utilization, Italy

## Abstract

Despite widespread childhood immunization programs, pneumococcal disease (PD) continues to be associated with significant clinical and economic burden worldwide. This retrospective study assessed the PD-related economic burden in children from the Veneto region of Italy following the introduction of a 13-valent pneumococcal conjugate vaccine (PCV13) to the Italian immunization schedule in 2010. Between 2010 and 2017, the annual incidences of pneumonia, acute otitis media (AOM), and invasive pneumococcal disease (IPD), as well as syndromic-disease-related episodes, declined. In our analysis of data from regional expenditure and healthcare resource utilization (HCRU) databases related to children < 15 years of age, we found that regional expenditures decreased between 2010 and 2017 for pneumonia (EUR 8.88 to EUR 3.59 million), AOM (EUR 3.78 to EUR 2.76 million), and IPD (EUR 1.40 to EUR 1.00 million). Despite reductions in PD-related expenditure following the introduction of PCV13, there continues to be an economic burden associated with PD in Veneto, Italy.

## 1. Introduction

*Streptococcus pneumoniae* is a pathogenic bacterium that causes pneumococcal disease (PD), with clinical manifestations that include pneumonia, acute otitis media (AOM), and invasive PD (IPD). IPD, which includes bacteremia, bacteremic pneumonia, and meningitis, can be fatal [1]. Although pneumococcal conjugate vaccines (PCVs) have been used extensively worldwide, PD continues to cause significant morbidity and mortality in children [2,3], in addition to being linked with a significant economic burden [4].

Immunization is the most effective public health strategy to address the clinical and economic burden of PD [5,6]. The 13-valent PCV (PCV13) was introduced in 2010 for pediatric vaccination and replaced the 7-valent PCV (PCV7) in the Veneto region of Italy. Veneto is Italy’s fifth-largest region, exceeding 4.9 million residents, of whom approximately 700,000 are < 15 years of age. The inclusion of PCV13 into the Veneto immunization schedule resulted in a decrease of 19.7% in the hospitalization of children < 4 years of age between 2011 and 2013 [7]. A Markov model determined that the introduction of PCV7 reduced IPD-related deaths from 129 to 19, from an estimated 4649 cases in the pre-PCV7 period; further reductions were seen following the introduction of PCV13 [8]. Between 2007 and 2014, there was a substantial reduction in the overall incidence of IPD (risk ratio [RR] = 0.4, 95% confidence interval [CI]: 0.3–0.5; *p* < 0.0001), especially in children < 5 years of age (RR = 0.3, 95% CI: 0.2–0.7; *p* = 0.0002) [9]. In Catalonia (a Spanish region with a similar population size to the Veneto region of Italy), AOM caused by PCV13 serotypes decreased from 65.5% to 48.4% between 2007 and 2013 among children ≤ 14 years of age [10].

Assessing the residual and vaccine-preventable burden of PD following the inclusion of higher-valency PCVs into national immunization programs can help determine their potential value in reducing PD-related health and economic burden. PD is associated with substantial direct medical costs and indirect costs (related to reduced productivity in parents and caregivers) [4,8,11]. For example, a decision-tree-based model used to assess healthcare resource utilization (HCRU) in Europe estimated that the total direct medical costs attributed to IPD in the pre- and post-PCV13 era were EUR 15.0 million and EUR 4.5 million, respectively [8]. In addition, the estimated direct medical costs per episode of IPD-related meningitis and bacteremia post-PCV7 introduction in Italy were EUR 8228 and EUR 10,874–11,789, respectively; in Spain, they were EUR 3239 and EUR 3786–4039, respectively [8].

To date, there have been few studies that highlight the economic burden of PD after the introduction of PCV13 in Italy, particularly in the Veneto region. Although Veneto had high pneumococcal vaccination coverage between 2007 and 2017, it has also been associated with increasing rates of IPD [12]. Therefore, the aim of this retrospective observational study was to improve our understanding of the incidence of pediatric PD and its economic burden in the early (2010–2013) and late (2014–2017) PCV13 periods in a well-defined region with a large pediatric population. We used clinical databases to define regional incidence and expenditure data at a patient level in order to determine the residual HCRU and direct costs associated with pneumonia, AOM, IPD and syndromic-disease-related episodes in children < 15 years of age.

## 2. Materials and Methods

### 2.1. Study Design

This was a retrospective, observational cohort study that assessed the incidence and economic burden associated with pneumonia, AOM, IPD and syndromic invasive disease in Venetian children < 15 years of age between 1 January 2010 and 31 December 2017. Two main databases were used: the Pedianet database and the Fascicolo Sanitario Regionale database.

The Pedianet database (http://www.pedianet.it, accessed on 1 April 2022) contains a network of more than 400 Italian family pediatricians (FPs) who use Junior Bit^®^ software (So.Se.Pe Srl, Padova, Italy). Among the 500 FPs in the Veneto region who use Junior Bit^®^, 52 are part of the Pedianet network and have approximately 50,000 children < 15 years of age enrolled in their practices.

Monthly Pedianet FP data collected from a centralized database in Padova are anonymized and validated. Included in the anonymized data are patient demographics, clinical characteristics (such as diagnoses using free-text or International Classification of Diseases, Ninth Revision, Clinical Modification [ICD-9-CM] codes), symptoms (or other medical observations related to the visits), ambulatory diagnostic exams, prescriptions, specialist visits, and diagnostic procedures. Patients consented to the inclusion of their clinical data in the Pedianet database; the inclusion of anonymized data from children required consent from parents or legal guardians.

The Fascicolo Sanitario Regionale database includes prescriptions, specialist visits, emergency room (ER) visits, and hospitalizations. ER visit and hospitalization data are linked to the Pedianet database for children with informed consent.

Using specific ICD-9-CM codes (Table S1) to search the databases described, we identified ER visits and hospitalizations associated with pneumonia, AOM, IPD and syndromic invasive diseases in children < 15 years of age. IPD caused by *S. pneumoniae*, or the presence of symptoms of *S. pneumoniae* infection (such as bacterial meningitis or unspecified sepsis), were included. Codes for pneumonia included conditions caused by any pathogen and excluded codes associated with IPD. Cases of pneumonia and AOM were additionally identified using free-text algorithms (Table S1) and were confirmed by the clinical data manager.

An episode was defined as at least one pneumonia-, AOM-, or IPD-related hospitalization, ER visit, or FP visit. New pneumonia or IPD episodes were defined by a period of 90 days without the occurrence of a pneumonia-related visit or IPD-related hospitalization, respectively, whereas new AOM episodes were defined by 14 days without an AOM-related medical visit.

### 2.2. Variables and Costs Considered

The HCRU and costs associated with pneumonia, AOM, IPD and syndromic diseases were grouped into FP ambulatory visits, antibiotic prescriptions, ambulatory diagnostic tests (e.g., pneumatic otoscopy or C-reactive protein test), specialist visits and examinations, ER visits, and hospitalizations. Costs per episode were calculated using the total costs divided by the number of episodes per year. Diagnostic-related group (DRG) tariffs for hospital admissions in Veneto were used to determine hospitalization costs [13]. Costs related to antibiotic prescriptions, ambulatory diagnostic tests, specialist visits, and specialistic exams were stratified for the different years, summed up, and divided by the number of episodes for the same year to estimate the average cost per single episode.

ER visits and hospitalization HCRU were estimated for each year, and the costs of the Veneto Region DRG tariffs were used for AOM and related complications and pneumonia [13]. Four possible AOM and pneumonia scenarios were used to assess ER costs (Table S2). Costs related to physicians’ and nurses’ person-time per episode were summed to these ER costs; for AOM episodes, half an hour of person-time for physicians and 3 h for nurses were considered (except for 25% of episodes, in which half an hour person-time for physicians and 11 h of person-time for nurses were considered). The cost of a physician consultation at its opportunity cost was estimated by dividing the capitation share per year (the sum of money per patient per year, paid in advance, for the delivery of primary care services [14]) by the yearly average number of visits, plus inflation, as measured by the Consumer Price Index (CPI) [15]. Similarly to FPs, pediatrician and nurse consultations were estimated by dividing the capitation share per year by the average number of working hours per week (Table 1). In the Veneto region, the mean salary for a physician working for the national health service in 2012 was approximately EUR 85,000 (EUR 42.34 per hour); the estimated person-time cost for a nurse was EUR 25.40 per hour [16]. Inflation, as measured by the CPI, was considered for each year [15] when assessing yearly physician consultation costs.

### 2.3. Statistical Analysis

Pneumonia, AOM, and IPD annual incidence rates, with the corresponding 95% CIs, were reported by year. The standardized regional incidence rate was calculated based on the direct standardization of Pedianet incidence data by age group for different years using regional population data.

We defined annual incidence rates (IRs) as the number of episodes per 1000 person years for pneumonia and AOM, and 100,000 person years for IPD and syndromic diseases, in the early (2010–2013) and late (2014–2017) PCV13 periods. The calculation of CIs and analyses to assess and compare trends of annual incidence rates in these periods were determined using previously published statistical methodologies [17].

In addition to the use of the Pedianet and Fascicolo Sanitario Project centralized databases, the Istituto Nazionale di Statistica (ISTAT) archive of statistics (produced by the Italian National Institute of Statistics) helped support the extrapolation of age and Veneto population data [18].

Regional expenditures for pneumonia, AOM, and IPD were calculated by multiplying the average cost per episode in 2017 with the standardized regional incidence rate using the formula: (total cost per episode × regional incidence rate × regional population) ÷ 1000. Regional expenditure was reported for all the outcomes of interest by year.

## 3. Results

From a total of 72,570 patients < 15 years of age enrolled in this study, 3927, 41,683, and 88 experienced pneumonia, AOM, and IPD and syndromic disease, respectively (Table 2). The annual number of pneumonia-, AOM- and IPD- and syndromic-disease-related episodes declined over the study period when comparing data from 2010 to 2017; similar reductions were recorded in annual IRs (Table 2). The Mann–Kendall test determined statistically significant monotonic decreasing trends for both pneumonia and AOM over the study period (−0.64, *p* = 0.026, and −1.00, *p* = 0.0005, respectively; Table 3); a non-statistically significant monotonic decreasing trend between 2010 and 2017 was recorded for IPD and syndromic diseases (−0.22, *p* = 0.46; Table 3). Despite recording numeric reductions for annual IRs in this study, interrupted time series (ITS) detected no statistically gradual or immediate changes in the early and late PCV13 periods for pneumonia, AOM, and IPD and syndromic diseases (Table 4).

HCRU data related to pneumonia, AOM, and IPD and syndromic disease episodes in children < 15 years of age are summarized in Table 5, Table 6 and Table 7. The total estimated cost per episode for individuals with pneumonia and AOM between 2010 and 2017 was highly variable, with a minimum observed in 2014 and a maximum in 2012 for pneumonia (EUR 494.21 and EUR 1048.20, respectively; Table 5); for AOM, a minimum was observed in 2010 and a maximum in 2013 (EUR 48.47 and EUR 50.12, respectively; Table 6).

The HCRU for pneumonia was primarily driven by hospitalizations, FP, and ED visits, of which the average costs per episode between 2010 and 2017 were EUR 714.63, EUR 29.00, and EUR 50.77, respectively (Table 5). The HCRU for AOM was driven by FP visits and antibiotic prescriptions, of which the average costs per episode were EUR 26.41 and EUR 8.71, respectively (Table 6). Furthermore, considerable reductions in the standardized regional incidence rates of pneumonia and AOM were recorded, with reductions from 13.30 to 5.48 and 112.88 to 82.28 per 1000 person years, respectively.

Following the introduction of PCV13, the total regional expenditures attributed to pneumonia, AOM, and IPD and syndromic invasive diseases decreased from 2010 to 2017 in children < 15 years of age (Table 5, Table 6 and Table 7). The annual total regional expenditure attributed to pneumonia decreased from approximately EUR 8.88 million to EUR 3.59 million (Table 5). Between 2010 and 2017, the annual total expenditure related to AOM decreased from approximately EUR 3.78 million to EUR 2.76 million (Table 6), while the annual total expenditure attributed to IPD and syndromic invasive disease decreased from approximately EUR 1.35 million to EUR 1.02 million (Table 7).

## 4. Discussion

Our results suggest that replacing PCV7 with PCV13 had a considerable impact on reducing the PD-related economic burden. Following the PCV7 era, the residual economic burden related to PD was substantial, and its replacement with PCV13 was associated with a decrease in annual PD-related costs [8]. A modelling study showed that, following the introduction of PCV7 in Europe, the direct medical costs attributed to IPD-causing serotypes contained in PCV7 were reduced from EUR 18.5 million to EUR 2.8 million [8]. While an increase in the direct medical costs attributed to serotypes unique to PCV13 was recorded in the PCV7 era, the subsequent replacement with PCV13 was associated with a substantial reduction of EUR 11.4 million to EUR 2.5 million [8]. Our analysis also showed reductions in total regional expenditures for PD following the introduction of PCV13 in the Veneto region.

Despite fluctuations in estimates of HCRU and the cost per episode for pneumonia and AOM, numeric reductions in the incidence of pneumonia, AOM, IPD and syndromic diseases may have been the main drivers of decreased expenditure. Our results are consistent with those from a previously published Italian study that concluded that the introduction of PCV13 was associated with the reduced incidence of AOM (1,576,211 versus 1,361,368 for unvaccinated and PCV13-vaccinated groups, respectively), and this may result in better health outcomes and decreased costs related to AOM treatment [19].

FP clinical diagnostic exams were the only AOM-related HCRU estimates that steadily increased between 2010 and 2017. These increases could be related to the updated guidelines from the Italian Society of Pediatrics, which recommended the use of pneumatic otoscopes for the management of AOM in children [20]. Furthermore, the unchanged use of antibiotic for AOM between 2010 and 2017 could be attributed to the ‘wait and see’ approach recommended by the Italian Society of Pediatrics [21].

Our results improve on the current paucity of data for HCRU and the costs associated with pneumonia, AOM, and IPD and syndromic disease in Italian children < 15 years of age. One of the last major studies to assess this was in 1999 and recorded a total direct medical cost for hospitalized acute lower respiratory tract infection of EUR 1435 [22]. Our study recorded estimated costs of EUR 966.34 and EUR 48.47 for treating each episode of pneumonia and AOM, respectively, considering costs either in the primary care setting or in the hospital setting. Another study reported total direct costs of EUR 163.94 per episode of physician-confirmed otitis media in Italy in 2007 [23]; our study recorded a cost per episode of EUR 48.47 in 2010. Estimations of costs were mostly similar, except for ER unit visit costs, which were five-fold higher than our estimate [23]. Moreover, a randomized controlled trial conducted between 2015 and 2018 in the Netherlands reported the mean total healthcare-related cost for AOM per patient to be approximately EUR 80, when excluding costs related to productivity losses suffered by parents [24]. Another strength of this study was that hospitalization costs related to the analysis of patient data were derived from linked regional healthcare databases. Data were collected from a variety of sources covering the full spectrum of healthcare costs, including hospitalizations, ER visits, primary and secondary specialty care visits, and prescriptions.

One important limitation of our study was that we did not control for co-morbid medical conditions in the study population, which could affect incidence and HCRU results. A study assessing the clinical and economic burden of IPD showed that costs and resource use per IPD-related episode differed between healthy and at-risk and high-risk children < 18 years of age who were immunocompetent with ≥ 1 chronic medical condition ($72,581, $259,382, and $1,029,071, respectively) [25]. While free text from clinical notes was evaluated by a clinical data manager to reduce outcome misclassification, our study was limited by the additional use of ICD-9-CM diagnosis codes to identify cases of pneumonia, AOM, and IPD and syndromic diseases. Clinical laboratory results confirming the pathogens causing these conditions were also not available for this study. Regarding IPD and syndromic diseases, we included both pneumococcal-specific ICD codes and ICD codes for related diseases in which *S. pneumoniae* could be a potential causative pathogen.

## 5. Conclusions

After the introduction of PCV13 in the Veneto region of Italy, the incidence of pneumonia, AOM, and IPD and syndromic disease decreased, with the largest reduction recorded for pneumonia-related episodes. This decline in incidence was associated with a corresponding decline in regional healthcare expenditures for pneumonia, AOM, and IPD between 2010 and 2017. While our results support the introduction of PCV13 for reducing the pediatric clinical and economic burden associated with PD, they also indicate that future PCVs will need to address the existing residual economic burden. The methodologies used in our study can be applied to other regions in Italy or countries that have similar clinical databases, to provide information on vaccine impact.

## Figures and Tables

**Table 1:** Per visit costs for FP, pediatrician, and nurse consultation, adjusted for inflation.

Year	CPI	FP Visit Costs Adjusted for Inflation (CPI)	Pediatrician Consultation Costs Adjusted for Inflation per Hour (CPI)	Nurse Consultation Costs Adjusted for Inflation per Hour (CPI)
		EUR	EUR	EUR
2010	1.52	23.97	39.91	23.95
2011	2.78	24.63	41.05	24.63
2012	3.04	25.38	42.34	25.40
2013	1.22	25.69	42.86	25.71
2014	0.24	25.75	42.96	25.78
2015	0.04	25.77	42.98	25.79
2016	−0.09	25.74	42.94	25.76
2017	1.23	26.06	43.47	26.08

CPI, Consumer Price Index; FP, family pediatrician.

**Table 2:** Pneumonia, AOM, and IPD annual incidence estimates in the pediatric population < 15 years of age (Pedianet 2010–2017).

Pneumonia				AOM			IPD		
Year	No. of Episodes (*N* = 3927)	Person Years	Annual IR × 1000 Person Years (95% CI)	No. of Episodes (*N* = 41,683)	Person Years	Annual IR × 1000 Person Years (95% CI)	No. of Episodes (*N* = 88)	Person Years	Annual IR × 100,000 Person Years (95% CI)
2010	559	43,692.02	13 (12–14)	5510	43,692.02	126 (123–129)	12	29,966.00	40.05 (17.39–62.70)
2011	534	47,609.77	11 (10–12)	5871	47,609.77	123 (120–126)	14	29,990.00	46.68 (22.23–71.13)
2012	408	50,643.46	8 (7–9)	5895	50,643.46	116 (113–119)	11	29,941.00	36.74 (15.03–58.45)
2013	454	52,192.36	9 (8–9)	5694	52,192.36	109 (106–112)	15	30,039.00	49.94 (24.67–75.20)
2014	772	52,805.74	15 (14–16)	5195	52,805.74	98 (96–101)	10	30,294.00	33.01 (12.55–53.47)
2015	403	52,392.12	8 (7–8)	4783	52,392.12	91 (89–94)	9	22,997.00	39.14 (13.57–64.70)
2016	389	52,184.01	7 (7–8)	4658	52,184.01	89 (87–92)	11	22,194.00	49.56 (20.28–78.85)
2017	276	51,348.38	5 (5–6)	4077	51,348.38	79 (77–82)	6	19,223.00	31.21 (6.24–56.18)

AOM, acute otitis media; CI, confidence interval; IPD, invasive pneumococcal disease; IR, incidence rate.

**Table 3:** Mann–Kendall analysis of annual IR of pneumonia, AOM, and IPD and syndromic diseases for children < 15 years of age.

	Mann–Kendall Test	
	Correlation	*p*-Value
Pneumonia	−0.64	0.0260
AOM	−1.00	0.0005
IPD	−0.22	0.4579

AOM, acute otitis media; IPD, invasive pneumococcal disease; IR, incidence rate.

**Table 4:** ITS analysis of annual IR of pneumonia, AOM, and IPD and syndromic diseases for children < 15 years of age.

	Early PCV13 Period ^†^		Late PCV13 Period ^‡^	
	Coefficient	*p*-Value	Coefficient	*p*-Value
**Pneumonia**				
Trend	−1.54	0.1191	−2.80	0.0003
Change in trend	NA	NA	−1.25	0.3201
Change in level ^§^	NA	NA	7.90	0.0380
**AOM**				
Trend	−5.80	0.0030	−5.88	<0.0001
Change in trend	NA	NA	−0.07	0.9558
Change in level ^§^	NA	NA	−5.69	0.1285
**IPD and Syndromic Disease**				
Trend	1.97	0.6342	0.50	0.8957
Change in trend	NA	NA	−1.47	0.7998
Change in level ^§^	NA	NA	−9.34	0.5036

^†^ The early PCV13 period was defined as the years between 2010 and 2013. ^‡^ The late PCV13 period was defined as the years between 2014 and 2017. ^§^ Immediate effect between early and late post-PCV13 periods. AOM, acute otitis media; IPD, invasive pneumococcal disease; IR, incidence rate; ITS, interrupted time series; NA, not applicable; PCV13, 13-valent pneumococcal conjugate vaccine.

**Table 5:** Pneumonia HCRU and related costs (*N* = 3926, Pedianet 2010–2017).

Year	FP Visit		Antibiotic Prescriptions		FP Clinic Diagnostic Exams		Specialist Visits		ED Visits		Hospitalizations		Total Cost Estimated per Episode (EUR)	Standardized Regional Incidence Rate × 1000 Person Years	Regional Population	Total Regional Expenditure ^†^ (EUR)
	Average No. per Episode	Cost per Episode (EUR)	Average No. per Episode	Cost per Episode (EUR)	Average No. per Episode	Cost per Episode (EUR)	Average No. per Episode	Cost per Episode (EUR)	Average No. per Episode	Cost per Episode (EUR)	Average No. per Episode	Cost per Episode (EUR)				
2010	1.21	29.05	1.02	14.09	0.17	1.85	0.25	6.42	0.36	57.98	0.26	856.95	966.34	13.30	690,641	8,876,340
2011	1.20	29.66	1.04	13.46	0.22	2.49	0.29	7.24	0.32	54.37	0.23	780.63	887.84	10.81	692,822	6,649,394
2012	1.12	28.49	0.98	11.00	0.26	2.95	0.22	5.53	0.39	66.95	0.28	933.28	1048.20	7.48	692,199	5,427,211
2013	1.12	28.90	1.03	12.28	0.19	2.23	0.25	6.17	0.26	45.75	0.19	627.98	723.31	10.16	694,514	5,103,865
2014	1.15	29.49	1.08	14.31	0.18	2.08	0.23	5.72	0.17	30.16	0.12	412.45	494.21	14.33	695,475	4,925,374
2015	1.14	29.35	0.99	12.12	0.28	3.10	0.28	6.69	0.25	44.37	0.18	612.04	707.67	7.47	689,172	3,643,166
2016	1.12	28.71	0.98	11.07	0.30	3.22	0.21	4.99	0.25	44.66	0.19	625.64	718.29	7.49	679,163	3,653,891
2017	1.09	28.33	0.95	10.41	0.39	4.29	0.15	3.67	0.35	61.89	0.26	868.05	976.64	5.48	670,057	3,586,136
Average	1.14	29.00	1.01	12.34	0.25	2.78	0.24	5.80	0.29	50.77	0.21	714.63	815.31	9.57	688,005.38	5,233,172

^†^ Standardized using total cost per episode in 2017 using the formula: (total cost per episode × regional incidence rate × regional population) ÷ 1000. ED, emergency department; FP, family pediatrician; HCRU, healthcare resource utilization.

**Table 6:** AOM HCRU and related costs (*N* = 41,683, Pedianet 2010–2017).

Year	FP Visit		Antibiotic Prescriptions		FP Clinic Diagnostic Exams		Specialist Visits		ED Visits		Hospitalizations		Hospitalization Because of AOM Complications		Total Cost Estimated per Episode (EUR)	Standardized Regional Incidence Rate × 1000 Person Years	Regional Population	Total Regional Expenditure ^†^ (EUR)
	Average No. per Episode	Cost per Episode (EUR)	Average No. per Episode	Cost per Episode (EUR)	Average No. per Episode	Cost per Episode (EUR)	Average No. per Episode	Cost per Episode (EUR)	Average No. per Episode	Cost per Episode (EUR)	Average No. per Episode	Cost per Episode (EUR)	Average No. per Episode	Cost per Episode (EUR)				
2010	1.05	25.08	0.93	9.75	0.18	3.07	0.04	0.77	0.77	1.71	0.0038	4.71	0.0016	3.38	48.47	112.88	690,641	3,778,699
2011	1.04	25.69	0.93	9.51	0.19	3.19	0.06	1.01	1.01	1.76	0.0039	4.85	0.0016	3.49	49.50	111.35	692,822	3,818,713
2012	1.05	26.57	0.91	8.56	0.22	3.42	0.04	0.80	0.80	1.81	0.0037	4.57	0.0015	3.27	49.00	106.93	692,199	3,626,825
2013	1.04	26.69	0.93	8.27	0.22	3.54	0.06	0.93	0.93	1.84	0.0042	5.14	0.0017	3.71	50.12	101.36	694,514	3,528,244
2014	1.04	26.84	0.90	8.07	0.23	3.55	0.05	0.93	0.93	1.84	0.0038	4.62	0.0013	2.84	48.68	92.70	695,475	3,138,425
2015	1.04	26.87	0.92	8.52	0.25	3.78	0.05	0.88	0.88	1.84	0.0038	4.71	0.0016	3.51	50.10	89.06	689,172	3,075,020
2016	1.03	26.64	0.91	8.47	0.25	3.74	0.05	0.90	0.90	1.84	0.0031	3.84	0.0020	4.30	49.73	90.20	679,163	3,046,484
2017	1.03	26.88	0.91	8.53	0.27	3.95	0.05	0.83	0.83	1.86	0.0031	3.77	0.0020	4.27	50.09	82.28	670,057	2,761,576
Average	1.04	26.41	0.92	8.71	0.23	3.53	0.05	0.88	0.88	1.81	0.00	4.53	0.00	3.63	49.46	98.35	688,005	3,346,748

^†^ Standardized using total cost per episode in 2017 using the formula: (total cost per episode × regional incidence rate × regional population) ÷ 1000. AOM, acute otitis media; ED, emergency department; FP, family pediatrician; HCRU, healthcare resource utilization.

**Table 7:** Costs related to IPD and syndromic invasive disease.

Year	IPD Incidence (100,000 Person Years)	Total Cost Estimated per Episode Based on DRG (EUR)	Regional Estimated Expenditure (EUR × 100,000 Person Years)	Regional Population	Total Regional Expenditure (EUR)
2010	40.05	4885.55	195,666.28	690,641	1,351,351
2011	46.68	4885.55	228,057.47	692,822	1,580,032
2012	36.74	4885.55	179,495.11	692,199	1,242,463
2013	49.94	4885.55	243,984.37	694,514	1,694,505
2014	33.01	4885.55	161,272.01	695,475	1,121,606
2015	39.14	4885.55	191,220.43	689,172	1,317,837
2016	49.56	4885.55	242,127.86	679,163	1,644,442
2017	31.21	4885.55	152,478.02	670,057	1,021,689
Average	40.79	4885.55	199,287.69	688,005	1,374,653

DRG, diagnosis-related group; IPD, invasive pneumococcal disease.

## Data Availability

The data used in this study cannot be made available in the manuscript; the Supplemental Files are in a public repository due to Italian data protection laws. The anonymized datasets generated and/or analyzed in the current study can be provided by the corresponding author on reasonable request, after written approval by the Internal Scientific Committee (info@pedianet.it).

## Supplementary Materials

The following supporting information can be downloaded at: https://www.mdpi.com/article/10.3390/children9091347/s1, Table S1: Search terms and diagnoses of pneumonia, AOM, and IPD and syndromic diseases: ICD-9 codes and free-text algorithms; Table S2: Possible AOM and pneumonia scenarios for the assessment of ER costs.
