# Supplementary material for: The Economic Burden of Pneumococcal Disease in Children: A Population-Based Investigation in the Veneto Region of Italy
Source: Children (Basel). 2022 Sep 3;9(9):1347. doi: 10.3390/children9091347 (PMC9498138; doi:10.3390/children9091347)
Supplement: Supplementary file 1 [file children-09-01347-s001.zip › children-1871173-supplementary.pdf]

Supplementary Materials

Table S1: Search terms and diagnoses of pneumonia, AOM, and IPD and syndromic diseases: ICD-9-CM codes and free text algorithms.

ICD-9-CM Codes	Description
**Pneumonia**	
480	Viral pneumonia
481	Pneumococcal pneumonia
482	Other bacterial pneumonia
483	Pneumonia due to other specified organism
484	Pneumonia in infectious diseases classified elsewhere
485	Bronchopneumonia, organism unspecified
486	Pneumonia, organism unspecified
487	Influenza with pneumonia
**AOM**	
381	Nonsuppurative otitis media and eustachian tube disorders
382	Suppurative and unspecified otitis media
384	Acute myringitis, unspecified
**IPD**	
038.2	Pneumococcal septicemia
038.0+041.2	Streptococcal septicemia + Pneumococcal infection

038.9+041.2	Unspecified septicemia + Pneumococcal infection
790.7+041.2	Bacteremia + Pneumococcal infection
038.0	Streptococcal septicemia
038.9	Unspecified septicemia
790.7	Bacteremia
320,1	Pneumococcal meningitis
320.2+041.2	Streptococcal meningitis+ Pneumococcal infection
320.8x/320.9 +041.2	Bacterial meningitis, unspecified + Pneumococcal infection
322.9 +041.2	Meningitis, unspecified + Pneumococcal infection
**Free Text Algorithm**	**Description**
**Pneumonia**	
""*olmon*"", ""*bp*"", ""*bn*"", ""*bm*""	Pneumonia
"oti*", "* oti*", "om*", and "* om*"	AOM

AOM, acute otitis media; ICD-9-CM, International Classification of Diseases, Ninth Revision, Clinical Modification; IPD, Invasive Pneumococcal Disease

Table S2. Possible AOM and pneumonia scenarios for ER cost assessment.

Disease Scenario	ER Cost (€)
**AOM**	
1% of episodes	113.40
3% of episodes	51.40
15% of episodes	45.50
81% of episodes	25.00
**Pneumonia**	
5% of episodes	163.30
5% of episodes	46.70
75% of episodes	70.65
25% of episodes	25.00
**IPD**	
URTI ad LRTI and patient aged <17 years of age	4741.19
Septic arthritis	4029.03
Septicaemia for patients aged <17 years of age	3563.78
Other diagnosis related to no specified infections	4490.49

AOM, Acute Otitis Media; ER, Emergency Room; €, Euros; LRTI, lower respiratory tract infection; URTI, upper respiratory tract infection
